# The Atypical Kinase RIOK3 Limits RVFV Propagation and Is Regulated by Alternative Splicing

**DOI:** 10.3390/v13030367

**Published:** 2021-02-26

**Authors:** Katherine E. Havranek, Luke Adam White, Thomas C. Bisom, Jean-Marc Lanchy, J. Stephen Lodmell

**Affiliations:** 1 Division of Biological Sciences, University of Montana, Missoula, MT 59812, USA; katie@fyrdiagnostics.com (K.E.H.); luke.white@umontana.edu (L.A.W.); jean-marc.lanchy@umontana.edu (J.-M.L.); 2 Department of Chemistry and Biochemistry, University of Montana, Missoula, MT 59812, USA; thomas.bisom@umontana.edu; 3 Center for Biomolecular Structure and Dynamics, University of Montana, Missoula, MT 59812, USA

**Keywords:** Rift Valley fever virus, MP-12, RIOK3, innate immune response, alternative splicing, morpholino oligonucleotide

## Abstract

In recent years, transcriptome profiling studies have identified changes in host splicing patterns caused by viral invasion, yet the functional consequences of the vast majority of these splicing events remain uncharacterized. We recently showed that the host splicing landscape changes during Rift Valley fever virus MP-12 strain (RVFV MP-12) infection of mammalian cells. Of particular interest, we observed that the host mRNA for Rio Kinase 3 (RIOK3) was alternatively spliced during infection. This kinase has been shown to be involved in pattern recognition receptor (PRR) signaling mediated by RIG-I like receptors to produce type-I interferon. Here, we characterize RIOK3 as an important component of the interferon signaling pathway during RVFV infection and demonstrate that RIOK3 mRNA expression is skewed shortly after infection to produce alternatively spliced variants that encode premature termination codons. This splicing event plays a critical role in regulation of the antiviral response. Interestingly, infection with other RNA viruses and transfection with nucleic acid-based RIG-I agonists also stimulated RIOK3 alternative splicing. Finally, we show that specifically stimulating alternative splicing of the RIOK3 transcript using a morpholino oligonucleotide reduced interferon expression. Collectively, these results indicate that RIOK3 is an important component of the mammalian interferon signaling cascade and its splicing is a potent regulatory mechanism capable of fine-tuning the host interferon response.

## 1. Introduction

Rift Valley Fever Virus (RVFV) is a mosquito-borne, negative sense RNA virus that belongs to the Phlebovirus genus within the Phenuiviridae family [1] that infects humans and livestock animals in Africa and the Arabian Peninsula. In humans, it can cause illness ranging from mild flu-like symptoms to hemorrhagic fever, blindness, liver disease, and death [2]. In livestock, RVFV infection results in a high mortality rate among young animals, and causes “abortion storms” in which nearly all infected pregnant animals will have aborted pregnancies [3]. Additionally, infection by RVFV in humans may lead to increased miscarriage rates [4]. It is currently categorized as a Category A pathogen and a select agent by the USA Centers for Disease Control, and no vaccines or drugs have been approved for human use in the general population. Therefore, it is important to understand the molecular virology of RVFV in order to develop effective antiviral therapeutic strategies. Here we describe a host factor, RIOK3, whose activity is antagonistic to propagation of RVFV MP-12, an attenuated vaccine candidate strain, and whose activity is modulated in cells via alternative splicing.

Alternative splicing (AS) is an important post- and co- transcriptional regulatory mechanism that influences the expression of more than 95% of genes in the human genome [5]. Genetic coding capacity is expanded by AS, making it one of the primary mechanisms generating diversity between the genetic code and the proteome [6]. AS allows the production of structurally diverse protein isoforms from a single gene, and can contribute to regulation of gene expression via its functional association with nonsense-mediated decay (NMD) [7,8]. 

Programmed changes in splicing patterns occur during embryonic development and maturation, as a consequence of disease, or as a response to changes in the external environment [9,10,11]. Prior work in our laboratory, as well as the work of others, has shown that widespread alteration in host alternative splicing patterns follow viral infection [12,13,14,15]. However, very little is known about how cellular splicing changes impact the outcome of viral infection. Here, we investigate a particular host gene, Rio kinase 3 (RIOK3), which we discovered to be alternatively spliced following RVFV MP-12 infection of HEK293 cells. RIOK3 was particularly intriguing since two previous studies suggested it is involved in the innate immune response that is important for controlling viral infection [16,17]. Furthermore, a recent report demonstrated that translation of RIOK3 is enhanced by an unusual mechanism involving methylation of the RIOK3 mRNA during flaviviral infection [18], which supports the ideas that RIOK3 is both an important component of the cellular response to infection, and a potential target for viral countermeasures against the cellular responses to infection.

MDA5 and RIG-I are RIG-I-like receptors (RLRs), which function as pattern recognition receptors (PRRs) that sense foreign RNA in the cytoplasm and transduce a signaling cascade to induce downstream type I interferon (IFN) production and the antiviral response [19]. RIOK3 was recently implicated in phosphorylation of the C-terminus of MDA5, which would inhibit MDA5 filament formation and prevent its interaction with MAVS, effectively quenching downstream signaling [17]. A separate report showed that RIOK3 acts as an adaptor protein that mediates the interaction between IRF3 and TBK1 downstream of PRR activation, which is critical for type I IFN induction following viral infection [16]. Thus, these two studies suggest that RIOK3 can act in dual roles, either stimulating or inhibiting innate immune activation. Notably, the experimental systems employed were different, and because different viruses activate distinct PRRs, the different roles for RIOK3 may depend on the pathway initially activated [20]. In any case, the clearly important roles of RIOK3 during viral infection warrants additional investigation.

In this study, we show that RIOK3 knockout led to increased RVFV MP-12 titer and mitigated downstream stimulation of IFN production. We also show that RIOK3 complementation in RIOK3 CRISPR/Cas9 knockout cells rescued production of IFN. Furthermore, in response to viral infection, cells produced alternatively spliced mRNA variants that were targeted by the nonsense-mediated decay machinery. Interestingly, transfection with the pan-RLR agonist, poly (I:C), and the RIG-I specific agonist, 3p-hpRNA, also triggered AS of RIOK3. Together, these data suggest that RIOK3 is an activator of the innate immune response and that its expression/activity is fine-tuned by alternative splicing in a likely auto-regulatory feedback loop.

## 2. Materials and Methods

### 2.1. Viruses, Cell Culture and Infections

The MP-12 vaccine strain of RVFV was kindly provided by Brian Gowen (Utah State University, Logan, UT, USA) and the rLuc RVFV reporter virus (also called delNSsrLuc MP-12), derived from MP-12 and containing the gene for Renilla luciferase in place of the viral NSs gene, was provided by Richard Elliott and Benjamin Brennan (University of St. Andrews, United Kingdom). Tacaribe virus (strain TRVL 11573) was a gift from Jack Nunberg (University of Montana), and the GFP-expressing adenovirus strain (admax-eGFP) and HCMV (strain TR) were gifts from Brent Ryckman (University of Montana). Sindbis virus (strain EgAr 339, NR-15695) was obtained through BEI Resources, NIAID, NIH. Manipulations of the viruses used in this study are compliant with both the Institutional Biosafety Committee at the University of Montana, Missoula, and NIH requirements in regard to their handling under BSL2 containment conditions.

HEK293 cells and Vero cells were cultured in Dulbecco’s Modified Eagle Medium (DMEM) with 10% fetal bovine serum and penicillin/streptomycin. For experiments using RVFV MP-12 or rLuc RVFV, 80–90% confluent HEK293 cells were washed with phosphate-buffered saline (PBS) and overlaid with virus at the specified multiplicity of infection (MOI) in DMEM without serum or antibiotics and incubated for one hour at 37 °C and 5% CO_2_. After incubation with virus the media was replaced with DMEM with 10% fetal bovine serum and penicillin/ streptomycin and the cells were maintained at 37 °C and 5% CO_2_ until time of harvest. For TCRV infections, 80–90% confluent HEK293 cells were washed with PBS and overlaid with TCRV inoculum in DMEM without serum or antibiotics sufficient to produce significant (~80%) CPE within 24 h of infection. After initial incubation with TCRV for 2 h, the media was replaced with DMEM with 10% fetal bovine serum and 1% penicillin/ streptomycin and the cells were maintained at 37 °C and 5% CO_2_ for 24 h. For ADV infections, 80–90% confluent HEK293 cells were washed with PBS and overlaid with ADV stock at an MOI of 10 (as titered by prior plaque assay) in DMEM without serum or antibiotics and incubated for one hour at 37 °C and 5% CO_2_, after which the media was replaced with DMEM with 10% fetal bovine serum and penicillin/ streptomycin and the cells were maintained at 37 °C and 5% CO_2_ for 24 h.

### 2.2. Plasmids and Cloning

The RIOK3 open reading frame was PCR amplified from Addgene plasmid #20618 (pWZL Neo Myr FLAG), which was a gift from William Hahn and Jean Zhao (Dana-Farber Cancer Institute, Boston, MA, USA) [21]. The RIOK3 ORF was cloned into phRL-CMV (Promega) between the XbaI and NheI restriction sites. The X2 variant was PCR amplified from cDNA obtained from infected HEK293 cells and was also cloned into phRL-CMV between the XbaI and NheI restriction sites. The pGL3-IFNB plasmid was obtained from Addgene, plasmid #102597 (IFN-Beta_pGL3), which was a gift from Nicolas Manel (Institut Curie, Paris, France) [22]. 

### 2.3. Transfection

Plasmid transfections were performed on HEK293 cells using Lipofectamine 2000 as per the manufacturer’s instructions (Thermo Fisher Scientific, Waltham, MA, USA). RIOK3 and GFP control FlexiTube siRNAs (Qiagen Germantown, Germantown, MD, USA) nos. Hs_RIOK3_4 and Hs_RIOK3_8) and HiPerFect Transfection Reagent were used for siRNA knockdown experiments in HEK293 cells according to the manufacturer’s instructions. Since HEK293 cells lack TLR3 expression for stimulation of innate immunity via poly (I:C), which stimulates both the MDA5 and the RIG-I pathways, and 3p-hpRNA, which stimulates only the RIG-I pathway, we transfected 1 μg/mL poly (I:C) (Tocris/BioTechne, Minneapolis, MN, USA) or 3p-hpRNA (Invivogen, San Diego, USA) into HEK293 cells using Lipofectamine 2000 according to the manufacturer’s instructions (Thermo Fisher Scientific). Morpholino oligos (Gene-Tools, Philomath, IN, USA) were transfected using Endo-Porter according to the manufacturer’s instructions (Gene-Tools). 

### 2.4. Western Blotting

Cells were lysed in radioimmunoprecipitation assay (RIPA) buffer with protease inhibitors (10 mM Tris-HCl pH 8.0, 140 mM NaCl, 1mM EDTA, 0.5mM EGTA, 1% Triton X-100, 0.1% sodium deoxycholate, 0.1% SDS). Clarified lysates were separated by SDS-PAGE on 10% polyacrylamide and wet transferred to PVDF. The membrane was blocked with 2.5% dry milk solution in Tris-buffered saline Tween 20 (TBST) at room temperature. Primary antibodies used include: Monoclonal M2 anti-FLAG (MilliporeSigma, Burlington, VT, USA), GAPDH loading control antibody MA5-15738 (Thermo Fisher Scientific), anti-RIOK3 SAB1406721 (Sigma). Following primary antibody incubation, the membrane was triple rinsed in TBST. HRP- conjugated secondary antibody used was anti-Mouse IgG peroxidase antibody produced in goat A2554 (Sigma). Following secondary antibody incubation, the membrane was triple rinsed with TBST. Chemiluminescent visualization of blots was carried out using Pierce ECL Western Blotting Substrate (Thermo Fisher Scientific) and a Fujifilm LAS 3000 imager.

### 2.5. Plaque Assays

HEK293 cells were treated with siRNA and infected with MP-12 (1.0 M.O.I.) as previously described. Supernatants were collected 24 hpi and titered via plaque assay. For plaque assays, Vero cells were grown to 90–100% confluence, washed with PBS, and overlaid with dilutions of virus in serum and antibiotic free DMEM, and infections were carried out as described above. Infected cells were overlaid with 3 mL of a 1:1 solution of 2% sterile low-melting agarose and 2× Eagle’s Minimal Essential Medium (2× MEM). Once solidified, plates were incubated at 37 °C and 5% CO_2_ for 6 days. After 6 days, agar overlays were discarded and the fixed cells were stained with crystal violet.

### 2.6. Luciferase Reporter Assays

For Renilla luciferase assays, HEK293 cells were either treated with RIOK3 or control siRNA, or transfected with either full-length RIOK3 cDNA or GFP expressing plasmids, and infected with rLuc RVFV (MOI of 1; measured by flow cytometry nucleocapsid protein positive staining cells at 24 hpi). Renilla luciferase activity was measured and assessed using the Renilla Luciferase Assay System (Promega, Madison, USA) according to the manufacturer’s instructions. For dual luciferase reporter assays, HEK293 cells were co-transfected with pGL3-IFNB firefly reporter and a 1:10,000 dilution of phRL-CMV Renilla control plasmid. 24 h post transfection, cells were infected with MP-12 (MOI of 1; titer measured by flow cytometry quantifying nucleocapsid protein positive cells at 24 hpi) or transfected with poly (I:C). At 48 hpi or 18 h post transfection, cells were assayed for dual luciferase activity using the Dual luc HT assay kit (GeneCopoeia, Rockville, MD, USA). 

### 2.7. CRISPR/Cas-Mediated Genome Editing

The expression of full length, X1, X2, or X1/X2 transcript isoforms from the RIOK3 gene in HEK293 cells was knocked-out in both alleles by CRISPR/Cas-mediated genome editing (RIO Kinase 3, gene ID: 8780, Ensembl: ENSG00000101782). Briefly, three distinct guide RNA were designed using the online resources of the Zhang Lab (https://zlab.bio/guide-design-resources). The concurrent use of three guide RNAs maximizes the chance of obtaining a knock-out cell clone. The best targets close to the start codon of the full length RIOK3 transcript were located in exons 3, 4, and 5 (exon 3 guide, plus strand, TACTTCCAGTGACCTTATGC-TGG; exon 4 guide, minus strand, GAGCTATCGCTGTCTTCATA-AGG; exon 5 guide, plus strand, ACCGGTTCCCACTCCTAAAA-AGG). Three DNA fragments (gBlocks, Integrated DNA Technologies, Coralville, IA, USA) bearing a single guide RNA sequence were each inserted in the pL-CRISPR.EFS.PAC plasmid, a gift from Benjamin Ebert (Addgene plasmid #57828; http://n2t.net/addgene: 57828; RRID: Addgene_57828) [23]. Sanger sequencing validated the exactness of the three individual plasmid constructs.

An equimolar mix of the three plasmids was then transfected in a fresh culture of HEK293 cells by the calcium phosphate precipitation method. The Cas9 enzyme is expressed in the transfected cells since the pL-CRISPR.EFS.PAC plasmid bearing the guide RNA genes also contains a Cas9 gene. After three days in cell culture, cells were counted with a hemocytometer, diluted, and dispensed in four 96-well dishes at a concentration of 0.5 cells per well, to maximize the chance of single cell well cloning. After one week of culture, all wells were visually analyzed and cells clusters were found in 90 wells out of a total of 384. Only 67 out of 90 positive wells were propagated further since they showed a single cluster of growing cells, whereas 23 wells showed two distinct cell clusters and were discarded (non-clonal). 

After several passages to amplify cell numbers, an aliquot of each clone was stored in liquid nitrogen (in 90% FBS and 10% DMSO), while another aliquot was processed for PCR-mediated RIOK3 allele analysis. Genomic DNA from each clonal population was extracted by an overnight incubation at 50 °C in lysis buffer (10 mM Tris-HCl pH7.5, 10 mM EDTA, 10 mM NaCl, 0.5% (*w*/*v*) sarkosyl, 1 mg/mL fresh Proteinase K) followed by phenol/chloroform extraction and ethanol precipitation. Genomic DNAs were digested by BamHI restriction enzyme to increase the screening efficiency during the PCR reactions. Each clonal genomic DNA was submitted to two distinct PCR reactions with a pair of primers located either in exons 3 and 4 or in exons 3 and 5 (exon3-F, CCAGTGACCTTATGCTG; exon4-R, TATCGCTGTCTTCATAAGGA; exon5-R, AATAAAGCCCTTTTTAGGAGT). The oligonucleotide primers were designed to have their 3′ ends bind to the region targeted by the guide RNA and, more precisely, to the nucleotides where most short deletions have been observed [24]. We thus expected that any change in the targeted region, even a small one, would negatively affect the PCR reaction outcome. Only five clones out of 67 remained negative for both PCR reactions, which was indicative that both alleles had been modified. Sanger sequencing and interpretation of the results showed that three clones out of five exhibited deletions within both alleles, leading to frame-shift induced appearance of premature stop codons in exons 3 and 4. These PTCs made the subsequent mRNAs good candidates for nonsense mediated decay. Even if these mRNAs escaped NMD and were translated, the severely truncated RIOK3 protein (predicted size 80–160 aa) would be missing the kinase domain that is assumed to be essential for its function. The lack of expression of any large RIOK3 protein species in the KO lines was confirmed by Western blotting using concentrated extracts from the three RIOK3-KO 293 cell clones.

### 2.8. RT-qPCR

Total RNA was extracted using the PureLink RNA Mini Kit (Thermo Fisher Scientific) or TRIzol (Thermo Fisher Scientific) and RNA was reverse transcribed using Superscript III First-Strand Synthesis SuperMix (Thermo Fisher Scientific) or Maxima H Minus Reverse Transcriptase (Thermo Fisher Scientific) with random hexamers according to the manufacturer’s instructions. qPCR was performed using the Applied Biosystems Step One Real-Time PCR System or the CFX Connect Real-Time PCR Detection System (Bio-Rad). RNA levels were normalized to GAPDH. Fold change in expression was calculated using the ∆∆CT method [25]. Primers used for qPCR are as follows: RIOK3 Exons 1/2 (F-GCCTTCATTCCCGAATGGATCTGGTAG, R-GCCAGCTGTTCACTCATTACATCAGCC), RIOK3 Exons 7/8 (Qiagen Quantitect Primer Assay), X2 variant (F-TGCCATCAAGAATGCAGAGA, R- TAACTGCCGCATCAAATGAA), GAPDH (Qiagen Quantitect Primer Assay), interferon beta (F- AAACTCATGAGCAGTCTGCA, R- AGGAGATCTTCAGTTTCGGAGG).

### 2.9. cDNA Cloning

Total RNA was extracted using the PureLink RNA Mini Kit (Thermo Fisher Scientific) from uninfected HEK293 cells and rLuc RVFV infected HEK293 cells 24 hpi. 600 ng of RNA was reverse transcribed using Superscript III First-Strand Synthesis SuperMix (Thermo Fisher Scientific) with random hexamers according to the manufacturer’s instructions. From the random hexamer cDNA pool, full-length (mRNA) RIOK3 cDNA was amplified using RIOK3-specific primers complementary to the start and stop codons sequences of the full-length RIOK3 ORF. Using this strategy, we examined only the fully processed full length mRNAs/cDNAs and not potentially transient splicing intermediates. RIOK3 cDNAs was cloned into the pDC316io expression plasmid using RIOK3-specific primers and transformed into E. coli. Collections of several individual clones, corresponding to mock- or RVFV MP-12 infected cells, were prepared by minipreparation of plasmid DNA and analyzed by Sanger sequencing. 

### 2.10. Cycloheximide Assays

HEK293 cells were treated with 100 μg/mL cycloheximide and total RNA was harvested at timepoints post-treatment. Total RNA was extracted using the PureLink RNA Mini Kit (Thermo Fisher Scientific) and treated with RQ1 DNase (Promega). 600 ng of RNA was reverse transcribed using Superscript III First-Strand Synthesis SuperMix (Thermo Fisher Scientific) with random hexamers according to the manufacturer’s instructions. RIOK3 species present in the cDNA were amplified by PCR using primers spanning several exons surrounding the alternatively spliced region (F- CCGGTTCCCACTCCTAAAAAGGGC, R- CCAGCATGCCACAGCATGTTATACTCAC) and CloneAmp HiFi PCR Premix (Takara, Mountain View, CA, USA). PCR products were run on a 1.8% agarose gel and stained with ethidium bromide. Fluorescence imaging was performed using a Fujifilm LAS-3000 imager.

### 2.11. Statistical Analysis

Statistical analyses were performed with GraphPad Prism 8 using a two-tailed unpaired Student’s *t*-test to assess significant differences between experimental treatments. Experimental error bars represent the standard error of the mean (SEM) (see figure legends). All qPCR data presented are representative of experiments including biological triplicates and technical duplicates. All data including RIOK3 knockout cells are representative of repeated experiments with at least two clonal cell lines.

## 3. Results

### 3.1. RIOK3 Is a Component of a Cellular Antiviral Pathway during RVFV MP-12 Infection

**Figure 1:**
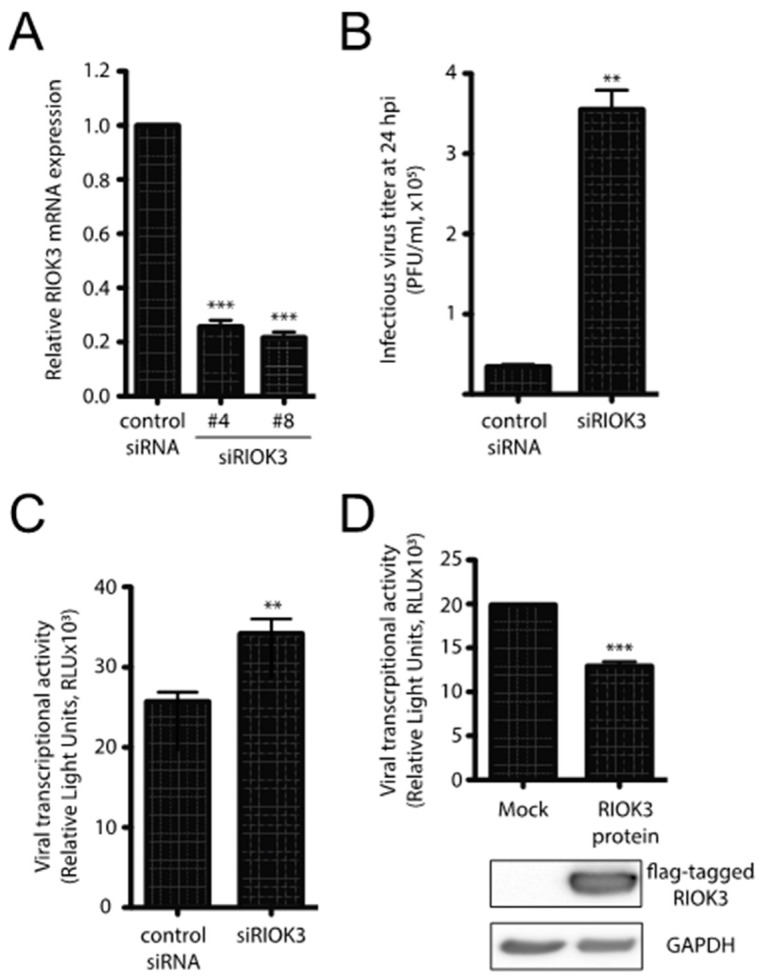
RIOK3 plays an antiviral role during RVFV MP-12 infection. (**A**) siRNA knockdown of RIOK3. HEK293 cells were transfected with RIOK3 (Qiagen nos. Hs_RIOK3_4 and Hs_RIOK3_8) or control siRNAs. Total RNA was extracted and RIOK3 mRNA was quantified by RT-qPCR. (**B**) Effect of RIOK3 knockdown on MP-12 titer. HEK293 cells were transfected with mock or RIOK3 siRNAs, then infected with RVFV strain MP-12. The viral titer in the supernatant at 24 hpi was quantified by plaque assay. (**C**) Effect of RIOK3 knockdown on rLuc RVFV transcriptional activity. HEK293 cells were transfected with siRNA targeted to RIOK3 or a negative control and infected with rLuc RVFV. The *Renilla* luciferase activity was quantified at 24 hpi and indicated the level of RVFV transcriptional activity. (**D**) Effect of RIOK3 overexpression on rLuc RVFV transcriptional activity. Cell lysates were prepared from HEK293 cells transfected with N-terminally FLAG tagged RIOK3 or mock transfected and protein levels were determined by Western blotting. Transfected cells were infected with rLuc RVFV and the level of *Renilla* luciferase activity was quantified at 24 hpi. In all panels, data are presented as mean +/− SEM of duplicate (**C**) or triplicate (**A**,**B**,**D**) biological replicates. Student’s *t*-test: *** *p* < 0.001, ** *p* < 0.01.

RIOK3 expression can be either necessary for or detrimental to viral propagation, depending on the virus [1,2,3]. To assess the importance of RIOK3 during infection, HEK293 cells were first transfected with control or RIOK3-targeting siRNAs and were infected 24 h later with RVFV MP-12. MP-12 is a commonly used attenuated strain of RVFV that allows for viral work to be completed under BSL2 containment conditions [4]. RIOK3 siRNA knockdown decreased RIOK3 mRNA by approximately 80%, which indicated that the transcription and the degradation of RIOK3 mRNA are relatively rapid (Figure 1A). At 24 hpi, newly made RVFV MP-12 particles were collected from the supernatants of the infected cells and the number of infectious particles was determined by plaque assay. Knockdown of RIOK3 resulted in a 10-fold increase of the number of infectious MP-12 particles produced (Figure 1B). This result indicated that normal expression of the RIOK3 gene is important to suppress the production of infectious particles [26]. 

To further analyze how the production of infectious particles could be affected by RIOK3 expression, we used a complementary reporter gene assay and mutant derivative of the MP-12 virus called rLuc RVFV [27] that harbors a *Renilla* luciferase open reading frame in place of the gene for the viral nonstructural protein NSs. NSs is a major virulence factor that plays several roles in RVFV pathogenesis and mitigates the host antiviral response by interfering with host transcription, PKR activation and IFNB promoter usage [28,29]. HEK293 cells were first transfected with control or RIOK3 siRNAs and infected 24 h later with rLuc RVFV. The siRNA-dependent decrease in RIOK3 expression correlated with an increase of rLuc gene expression (Figure 1C). Since the rLuc gene is both virally encoded in the mutant virus, and its expression depends on an efficient viral transcription by L and N viral products, the results in Figure 1C suggest that RIOK3 decreases the expression of virally encoded luciferase in infected cells. This model is further corroborated by the fact that overexpression of exogenous FLAG-tagged RIOK3 followed by infection with rLuc RVFV yielded a *decrease* in virally encoded Renilla luciferase protein production as measured by luciferase activity (Figure 1D). Taken together, these results indicate that RIOK3 expression correlates with diminished viral replication and protein expression, and thus functions in an antiviral capacity during RVFV infection.

### 3.2. RIOK3 Is Involved in the Activation of Type I IFN Response

**Figure 2:**
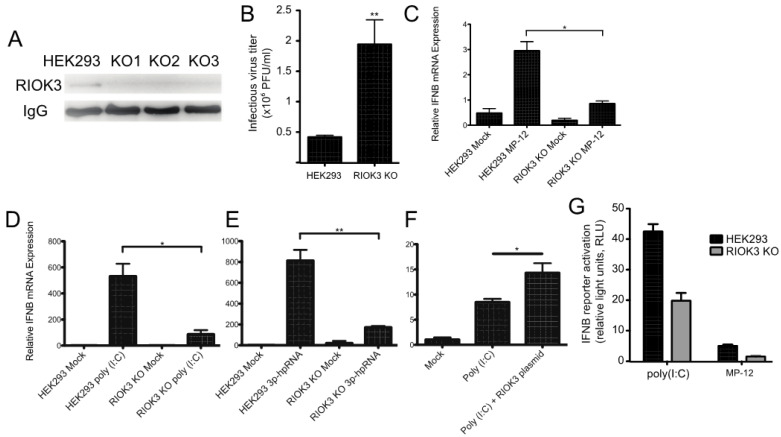
RIOK3 is involved in the signal transduction of the type I IFN response. (**A**) HEK293 cells and three RIOK3 KO cell lines were screened for RIOK3 protein expression by IP-Western blot. (**B**) HEK293 cells and RIOK3 KO cells were infected with MP-12 (MOI 1.0). The viral titer in the 24 hpi supernatant was quantified by plaque assay. (**C**,**D**) RT-qPCR targeting IFNB was performed on RNA from HEK293 cells and RIOK3 KO cells infected with MP12 (**C**) or treated with poly (I:C) (**D**) or 3p-hpRNA (**E**) for 18 hours. (**F**) RT-qPCR targeting IFNB was performed on RIOK3 KO cells transfected with a GFP (mock) or RIOK3 expression plasmid and treated with poly (I:C) 18 hours later. (**G**) Effect of RIOK3 KO on IFNB promoter activation. HEK293 cells and RIOK3 KO cells were co-transfected with pGL3-IFNB firefly reporter and phRL-CMV renilla control. Cells were stimulated with poly (I:C) or infected with MP-12 and the dual luciferase signals were measured after 18 or 48 h, respectively. In panels B–F, data are presented as mean +/− SEM from three biological replicates. Western blots in panels A is representative of duplicate experiments. Student’s t-test: ** *p* < 0.01, * *p* < 0.05.

To further investigate the role(s) and significance of RIOK3 in the antiviral response, a RIOK3 CRISPR knockout (KO) cell line was generated (see Materials and Methods). Sequence analysis of genomic PCR amplicons of CRISPR targeted regions revealed frameshift mutations and deletions in both chromosomal copies RIOK3 in several clones and IP-western analysis confirmed the absence of RIOK3 in three different CRISPR KO cell clones (Figure 2A). As a functional screen, RIOK3 KO and normal cells were infected with MP-12 and the titers of the newly synthetized viral particles released from the infected cells were analyzed by plaque assay, as described for Figure 1B. Similar to the siRNAs-mediated knockdown of RIOK3 expression, RVFV MP-12 replication in the infected RIOK3 KO cells led to a reproducible increase of the viral titer when compared to normal cells (Figure 2B). 

Previous studies suggested that RIOK3 is involved in the transmission of activation signals from RNA-specific pattern recognition receptors to the promoters of interferon-specific genes [1,2,3]. To test whether RIOK3 was involved in IFN expression during RVFV infection, we performed RT-qPCR targeting IFNB mRNA in RVFV-infected HEK293 cells and RIOK3 KO cells. We found that IFNB expression was significantly diminished in the RIOK3 KO cell lines (Figure 2C). Next, we wanted to determine which upstream factors this IFNB expression decrease could be attributed to. To test this, we transfected poly (I:C)- or 3p-hpRNA into RIOK3 KO cells and compared IFNB expression to normal stimulated HEK293 cells. Poly (I:C) is a double stranded RNA analog that is known to elicit cellular responses similar to those that occur during certain RNA virus infections by activating both the MDA5 and RIG-I pathways [30], and 3p-hpRNA is a specific RIG-I agonist comprised of an 87-nt hairpin [31]. We found that the RIOK3 KO cells had a significant decrease in the amount of total interferon beta (IFNB) mRNA when stimulated in two independently isolated clones of the RIOK3 KO, representative data are presented in Figure 2D,E. We then complemented RIOK3 KO cells with an expression plasmid encoding full-length RIOK3 protein to assess whether restoring expression of RIOK3 could rescue IFNB expression. We treated plasmid-transfected cells with poly (I:C) and tested IFNB via qRTPCR, and found that IFNB expression was increased when RIOK3 was added back to cells via plasmid (Figure 2F). These data indicate that RIOK3 acts downstream of RIG-I activation and upstream of IFNB expression.

In parallel, an IFNB reporter plasmid was transfected in either HEK293 or RIOK3 KO cells to measure the level of IFNB promoter activation (Figure 2G). Following transfection, the cells were stimulated by either poly (I:C) transfection or MP-12 infection and the level of IFNB promoter activity was measured by luciferase assay. The RIOK3 KO cell lines exhibited significantly decreased activation of the reporter plasmid following both types of stimulation, indicating that RIOK3 is involved in the signal transduction during IFNB promoter activation at a step either concomitant with or preceding IRF3 translocation into the nuclear compartment. 

### 3.3. RNA Virus Infection and Poly (I:C) Trigger RIOK3 Alternative Splicing

Our prior work showed that RIOK3 alternative splicing increased during RVFV MP-12 infection [10]. The importance of studying the population of RIOK3 splicing isoforms is underscored by the fact that translation of X1 or X2 splice variants described below would produce truncated RIOK3 protein isoforms missing most of the structural kinase domain. There are several mRNA isoforms described in GenBank; in addition to the canonically spliced RIOK3 transcript (NM_003831), which we call “full-length” here to indicate that it incorporates the largest exon 8 (see Figure 5) and does not lead to incorporation of premature termination codons, two other near full-length splice variants are documented: XM_011526242 (X1 variant), XM_011526243 (X2 variant), which affect splicing in exons 7 and 8, respectively [32]. The X1 transcript corresponds to full-length RIOK3 but with exon 7 skipped altogether during splicing. The X2 variant, whose abundance increased during MP-12 infection, uses an alternative 5’ splice site in exon 8. The X1 event also appeared as a differentially spliced transcript occurring during infection, but it was below threshold parameters so it was not considered a statistically significant event [10]. 

**Figure 3:**
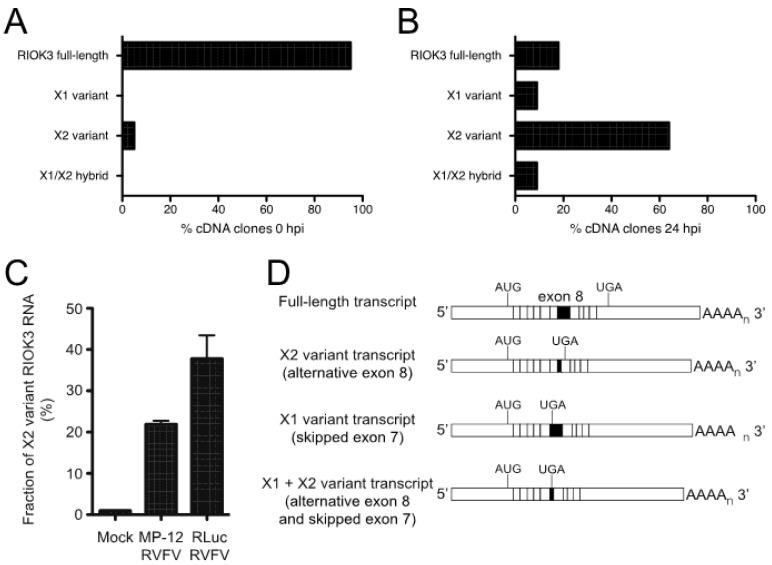
RVFV infection induces alternative splicing of RIOK3 transcripts. Distribution of RIOK3 splicing isoforms in either mock-infected (**A**) or rLuc RVFV infected (**B**) HEK293 cells. Total RNA was purified from mock- or virus-infected cells, reverse transcribed/PCR amplified into mRNA-length cDNAs, and cloned in a plasmid. From each sample, 40 individual clones were sequenced and categorized as full length, X1, X2, or X1/X2 hybrid. (**C**) Prevalence of RIOK3 X2 alternative splicing in infected cells. HEK293 cells were infected (MOI 1.0) with either MP-12 or rLuc RVFV and total RNA was extracted after 24 h. The fraction of X2 variant alternative splicing was quantified by RT-qPCR using primers specific to detect the canonical and X2 isoforms at exons 8 and 9, and not necessarily in full length poly-adenylylated mRNAs, which were characterized in panels (**A**,**B**). Data in panel **C** is presented as mean +/− SEM from triplicate experiments. (**D**) Schematic of the different splicing patterns observed. X2 employs a cryptic splice donor site within exon 8, resulting in a shortened exon 8 and a new stop codon in exon 9. X1 skips exon 7 entirely. Some transcripts contained both X1 and X2 type alternative splicing.

To capture the different RIOK3 transcripts present during rLuc RVFV infection and confirm the presence of X2 and X1 variants, cDNAs were produced and sequenced from the RNA lysates of both mock- and rLuc RVFV-infected cells. We amplified cDNA with primers in the first and last exons of RIOK3 to enrich for only full-length mRNAs and not partially processed fragments and cloned them into a plasmid. Individual clones were purified and sequenced. The population of large RIOK3 transcripts in uninfected cells largely consisted of full-length RIOK3 mRNA (NM_003831), with a small proportion of the RIOK3 X2 variant (Figure 3A). However, the cDNA clones from infected cells correspond to mostly alternatively spliced species, with only a small proportion of full-length RIOK3 (Figure 3B). An X1/X2 hybrid variant, the product of both exon 7 skipping and exon 8 truncation, was also detected in infected cells. Since we detected a large increase of the RIOK3 X2 variant when sequencing fully processed mRNA-length RIOK3 isoforms, RT-qPCR with primers flanking the exon 8 was used to compare the levels of X2 alternative splicing events in cells infected with either MP-12 or rLuc RVFV particles at a multiplicity of infection (MOI) of 1 (MOIs were defined by the percentage of cells expressing the viral nucleocapsid protein in the first 24 h post infection as measured by flow cytometry). Relative to mock infected cells, the X2 variant increased by ~22 fold by 24 hpi in cells infected with MP-12 (Figure 3C). In rLuc RVFV infected cells, the X2 splicing variant increased by ~38 fold by 24 hpi (Figure 3C). The greater increase in RIOK3 X2 variant alternative splicing during rLuc RVFV infection is probably related to the lack of the major virulence factor NSs that effects host transcriptional shutoff. Thus, the rLuc infected cells produce more RIOK3 transcripts early in infection, and alternative splicing ensues, promoted either by cellular regulatory mechanisms or by the virus to blunt the innate immune response. The fact that X2 alternative splicing occurs with the NSs-defective rLuc virus indicates that NSs is not the root cause of this alternative splicing.

**Figure 4:**
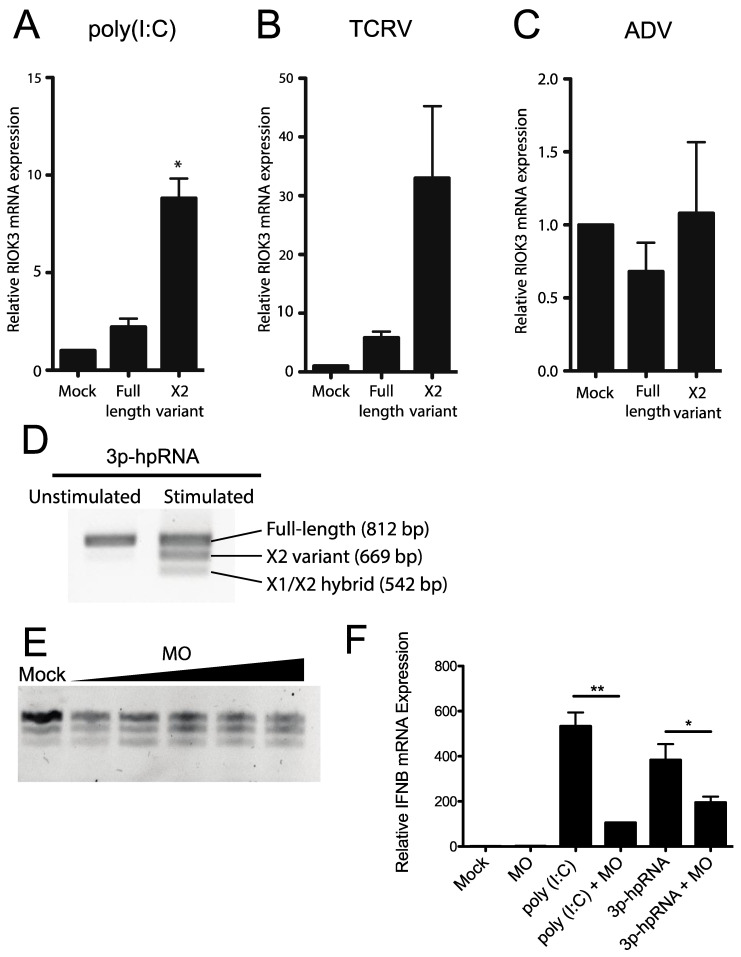
Activation of cytosolic innate immune RNA sensors, but not DNA sensors induces RIOK3 X2 variant alternative splicing, and RIOK3 splicing is vital for IFN expression. HEK293 cells were transfected with poly (I:C) (**A**), or infected with either the RNA virus Tacaribe (TCRV) (**B**), or the DNA virus adenovirus (ADV) (**C**). Total RNA was harvested 24 h post transcription or infection and RT-qPCR was used to quantify the relative fraction of X2 variant and full-length canonically spliced RIOK3 species. Data are presented as mean +/− SEM based on duplicate experiments. Student’s *t*-test: * *p* < 0.05. (**D**) HEK293 cells were transfected with 1 μg/mL 3p-hpRNA. RT-PCR targeted the region spanning RIOK3 exons 5–10, followed by agarose gel electrophoresis. Splicing isoforms are indicated. (**E**) Morpholino oligonucleotide targeting the canonical exon 8 splice donor site of RIOK3 pre-mRNA was transfected into HEK293 cells in increasing concentration (2–10 μM) for 18 h. RNA was processed via RT-PCR and run on agarose gel. (**F**) RT-qPCR was performed to measure the expression of IFNB mRNA after 18 h MO (8 μM) transfection and subsequent stimulation by either poly (I:C) or 3p-hpRNA. Data is presented as mean +/− SEM from triplicate experiments. Student’s *t*-test: ** *p* < 0.01, * *p* < 0.05.

To determine whether RVFV MP-12 infection presents a unique circumstance under which RIOK3 alternative splicing is activated, other viral or viral-like stimuli were tested for their ability to prompt an increase in RIOK3 X2 variant alternative splicing. Transfection of HEK293 cells with poly (I:C) increased the fraction of RIOK3 X2 variant RNA (Figure 4A). Additionally, transfection of 3p-hpRNA, a short double-stranded RNA that specifically stimulates RIG-I (and not MDA5), caused an increase in RIOK3 X2 mRNA (Figure 4D). Similarly, infection of HEK293 cells with Tacaribe virus (TCRV), a New World Arenavirus with single-stranded negative sense RNA genome, increased the fraction of RIOK3 X2 variant RNA (Figure 4B). In contrast, infection with Adenovirus, a double-stranded DNA virus, did not promote RIOK3 X2 variant alternative splicing (Figure 4C). Similar trends were found in preliminary studies with Sindbis virus (EgAr339), a positive sense single-stranded RNA alphavirus that also caused RIOK3 alternative splicing, and with human cytomegalovirus HCMV (strain TR), a large double-stranded DNA herpesvirus that did not trigger RIOK3 alternative splicing [33]. These results suggest a relationship exists between RIOK3 alternative splicing and activation of the viral RNA sensors of the interferon response pathways, particularly RIG-I, during infection with the RNA viruses and treatments tested. 

### 3.4. Canonical RIOK3 mRNA Splicing Is Vital for IFN Activation

We next tested whether the RIOK3 X2 splicing event had an effect on IFNB production. We used a morpholino oligonucleotide (MO) (Gene Tools, LLC, Philomath, OR, USA) to occlude the RIOK3 FL splice site while treating cells with poly (I:C) or 3p-hpRNA. Morpholino oligos are highly stable nucleic acid mimics that can be used to occlude splice junctions in pre-mRNAs without eliciting degradation of the RNA by cellular RNaseH activity [34,35]. We hypothesized that transfection of the appropriate MO would force cells to express more X2 RIOK3 mRNA isoform without activating the RLR pathways. We further hypothesized that increased expression of the X2 isoform would result in decreased expression of full-length RIOK3 protein, affecting downstream IFNB mRNA expression. First, to confirm that X2 expression is increased upon MO treatment, we transfected cells with increasing concentrations of MO and performed RT-PCR followed by gel electrophoresis (Figure 4E). The gel was visualized on a Fuji LAS-1000 imager and the band intensities were quantified using ImageJ. Even at the lowest concentration (2 μM) of transfected MO, we observed a marked increase in X2 isoform mRNA expression that accompanied decreased abundance of canonically spliced RIOK3 mRNA, from 24% alternatively spliced isoforms in mock-transfected cells to 45% at 2 μM MO. This percentage increased with increasing MO dosage up to 60% alternatively spliced at 10 μM (Figure 4E). Next, we used qRT-qPCR to quantify IFNB mRNA expression on poly I:C stimulated cells that were pre-treated with the MO. We observed that IFNB mRNA expression was reduced as a result of the MO treatment (Figure 4F). This indicates that the RIOK3 alternative splicing event can mitigate the cellular innate immune response, which may be important to limit an excessive or prolonged inflammatory/antiviral response. 

### 3.5. RIOK3 X2 Variant RNA Transcripts Are Substrates for Nonsense-Mediated Decay

**Figure 5:**
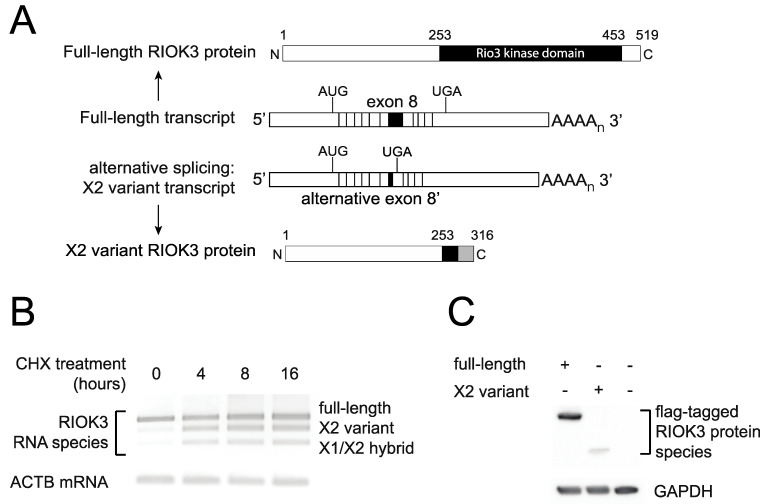
RIOK3 X2 variant mRNA encodes a truncated protein product and is targeted by nonsense-mediated decay (NMD). (**A**) Schematic of the normal and X2 variant alternative splicing on the precursor RIOK3 RNA transcript together with a representation of the full-length and X2 variant RIOK3 protein isoforms. (**B**) Alternatively spliced RIOK3 mRNA is efficiently degraded by nonsense mediated decay. HEK293 cells were mock treated or treated with cycloheximide (CHX) for the times indicated. Total RNA was harvested and subjected to RT-PCR using primers flanking the spliced exons. PCR products were run on agarose and stained using ethidium bromide. As CHX inhibits NMD, X2 and X1/X2 products are preserved with treatment. Actin B was used as a control. (**C**) Expression of RIOK3 full-length and X2 variant proteins from transfected expression plasmids. RIOK3 full-length and X2 constructs were transfected into HEK293 cells and expression was analyzed by Western blot. The presence of the same FLAG tag at the N-terminus of both proteins allowed us to compare their expression efficiencies.

Although no structural data currently exist for RIOK3, sequence homology based on the known RIOK1 functional domains allows for prediction of two domains: an N-terminal domain that is divergent from other RIO family proteins and whose function is largely undetermined, and a highly conserved C-terminal RIO kinase domain [36]. In the case of the RIOK3 X2-variant, the alternative splicing results in a frameshift that introduces a downstream premature termination codon (PTC) in exon 9. This leaves a much smaller open reading frame that encodes a truncated RIOK3 protein lacking most of the kinase domain (Figure 5A). Furthermore, the introduction of a PTC renders these transcripts canonical candidates for nonsense-mediated decay (NMD), since a termination codon greater than 50 nucleotides upstream of the final exon junction is considered to be premature and triggers NMD in mammalian cells [37]. To test whether RIOK3 X2 variant RNA transcripts are targeted by the NMD machinery, cells were treated with cycloheximide, a translation inhibitor and commonly used probe of NMD activity [38]. Cycloheximide is an effective NMD inhibitor because NMD relies on functional ribosomes and active translation. RNA was isolated from treated and untreated cells, reverse transcribed to cDNA and PCR amplified with primers flanking the alternatively spliced exons. Two bands appeared over time in cycloheximide treated cells that corresponded to the X2 and X1/X2 hybrid variants (Figure 5B). The identity of these bands was verified by sequence analysis. These results demonstrate that the alternatively spliced X2 variant RIOK3 transcript is a NMD substrate. 

In our system, despite the documented abundance of the X2 variant RIOK3 transcript, we could not detect an endogenously expressed truncated protein corresponding to its translation using commercially available antibodies, suggesting that either the mRNA is rapidly degraded or that the truncated protein is poorly detectable or unstable in cells. We cloned both full-length and X2 variant RIOK3 cDNAs into a mammalian expression construct with a FLAG tag on their N-terminus. Transfection of stoichiometrically equivalent amounts of either full-length or X2 variant FLAG-tagged constructs into HEK293 cells showed detectable amounts for both FLAG-tagged proteins (Figure 5C). However, the weaker band intensity of the X2 variant protein suggests either that the protein expression from the X2 variant RIOK3 construct was much less robust than the one from the full-length RIOK3 construct or that the shorter protein is turned over rapidly. In summary, it is likely that the X2-spliced mRNA variant product of RIOK3 is targeted for decay by the NMD machinery, and translation of any X2 variant mRNA that survives NMD degradation may produce an unstable and catalytically inactive truncated version of the full-length RIOK3 kinase protein owing to its truncated C-terminal region.

## 4. Discussion

Upon infection by any virus, mammalian cells take immediate steps to limit viral success, and concurrently the virus initiates measures to limit the cell’s antiviral responses. In prior work, we discovered that soon after infection with RVFV MP-12, transcription of the kinase, RIOK3 is upregulated, and curiously, the splicing pattern of the RIOK3 mRNA changes rapidly as well. In the work presented here, we demonstrate that RIOK3 expressed in its canonically spliced form is important for interferon expression, an important antiviral response. We further demonstrate that expression of the alternatively spliced isoform of RIOK3 does not contribute to the robust interferon response. We propose that the alternative splicing of RIOK3 mitigates the IFN response, and that this alternative splicing is programmed by the cell to prevent an excessively prolonged interferon response, or that this mechanism is hijacked by the virus early in infection to blunt the antiviral response to infection, or both. 

Regulation of alternative splicing is a complex process that is not based on a clearly understood set of rules. Rather, it is a layered regulatory phenomenon in which splice site selection is determined by a variety of cis- and trans-factors acting in concert [39]. The picture becomes even more complex during viral infection, where the host-pathogen dynamic strongly influences cellular metabolism. Alternative splicing patterns during viral invasion are often a combination of a programmed host response to counteract the infection as well as viral efforts to interfere with the host splicing machinery [40,41].

Our prior transcriptome profiling study of RVFV MP-12-infected cells indicated that RIOK3, a host factor recently characterized as an important player in the host antiviral response, is alternatively spliced during infection. This splicing event was particularly intriguing since work performed by Feng and colleagues illustrated that knockdown of RIOK3 during infection with MHV-68 herpesvirus and influenza A virus (IAV, strain A/WSN/33) in HEK293T cells led to an increase in viral titer of 5-fold and 15-fold, respectively [16]. They further showed that RIOK3 is required for induction of type I IFN after challenge with Sendai Virus (SeV) or poly(I:C). Their model suggests that RIOK3 mediates the interaction between IRF3 and TBK1, which is required for IRF3 dimerization, subsequent translocation into the nucleus and production of type I IFN and transcription factors NF-κB and AP-1 [42]. In contrast, work by Takashima and colleagues suggested that RIOK3 phosphorylates the pattern recognition receptor MDA5 at Ser-828, which hinders MDA5 activation in uninfected and virus-infected HEK293 cells, thereby attenuating the antiviral response [17]. Specifically, they showed that RIOK3 knockout enhanced the type I IFN response after measles virus (MV) infection. MDA5 activation is triggered by RNA elements from certain viruses, like measles, polio and encephalomyocarditis viruses [43,44,45], while RIG-I is the PRR that activates the IFNB pathway during infection by a distinct subset of negative sense RNA viruses, including IAV, SeV and RVFV [19,20,42,46]. Furthermore, Gokhale and colleagues reported that RIOK3 is required for productive infection of Dengue virus and Zika virus, but is inhibitory towards hepatitis C virus [18]. Differential responses to particular types of viruses could explain the apparent discrepancy between these studies but they nevertheless suggest dual functions for RIOK3 within the same or parallel pathways culminating in production of interferon. 

In the present study, we show that RIOK3 acts in an antiviral capacity during RVFV MP-12 infection and plays a critical role in activation of IFN signaling. Knockdown or knockout of endogenous RIOK3 led to enhanced viral replication and decreased activation of type I IFN signaling, and re-addition of RIOK3 to knockout cells rescued IFN signaling. The increase in viral replication after knockdown/knockout is notable since cellular mutations that diminish cellular vitality can also diminish virus propagation. Several other genes whose disruption is linked to higher virulence or disruption of innate immune pathways have been identified in previous studies (e.g., [47,48]). Our observation that overexpression of full length RIOK3 makes wild type cells modestly more resistant to infection is consistent with its role in a pathway(s) in which both the abundance and the cellular activation of the components of the pathway are essential to observe the response. Thus, overexpression of RIOK3 would be expected to aid in the cellular antiviral response, but other components of this pathway that are not overexpressed would become rate-limiting. 

This report shows that RIOK3 is a critical component of the IFN signaling pathway triggered by RVFV MP-12 infection and by treatment with poly (I:C) and 3p-hpRNA. As the effect of knocking out RIOK3 is very pronounced when treating cells with 3p-hpRNA, we propose that RIOK3 is component of the RIG-I pathway. 

The marked change in the splicing pattern of RIOK3 mRNA upon infection or treatment with poly (I:C) or 3p-hpRNA is intriguing. Interestingly, the alternatively spliced X2 variant RIOK3 transcript was represented in spliced EST samples collected from both humans and mice (see Table 1), suggesting a conserved functional role outside of the context of infection. Here, we show that altering the abundance of RIOK3 isoforms to favor X2 through MO treatment caused a significant decrease in IFNB mRNA expression, implying that this alternative splicing event represents a mechanism to control the IFNB response. It is curious that innate immune activators would simultaneously cause a splicing event that deactivates innate immunity. A plausible explanation is that the AS occurs to prevent excessive innate immune response, which would be detrimental to the cell and surrounding tissue in vivo. A study closely measuring the temporal regulation of splicing and immune activation will be important to distinguish these seemingly disparate effects. 

**Figure 6:**
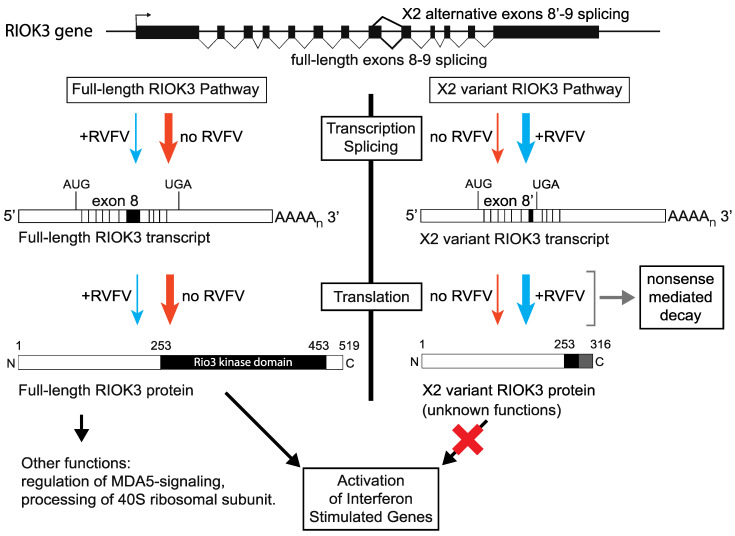
Relationship between RIOK3 splicing products, RVFV MP-12 infection, and innate immune activation. The human RIOK3 gene is represented at the top of the panel with its 13 exons. The normal RIOK3 protein with a complete kinase domain is called full-length and produced by translation of a canonically spliced transcript encompassing the 13 exons (left side of figure). Alternative 5′ splice donor usage shortens exon 8 and results in the X2 variant mRNA. Translation of the X2 mRNA would produce a truncated RIOK3 missing most of the kinase domain (right side of figure). Notably, the X2 RNA contains an exonic premature termination codon making it a canonical substrate for nonsense-mediated decay and rapid degradation. The effects on RIOK3 gene expression during MP-12 infection (blue arrows) compared to uninfected cells (orange arrows) are shown. The magnitude of the effect is represented by the thickness of each arrow. It is not known if the X2 variant RIOK3 protein is expressed or stable, or if it possesses any function. By contrast, the full-length RIOK3 protein has been suggested to be involved in several diverse cell functions and pathways (see text), thus the relative expression levels of full length and X2 variants of RIOK3 can have strong effects on cellular functions including the antiviral response.

How does AS regulate RIOK3 function? The two non-mutually exclusive possibilities are that AS simply reduces the intracellular level of functional RIOK3 protein, or that the protein expressed from the AS transcript has inhibitory properties itself. The putative protein product of RIOK3 X2 variant is truncated at the C-terminal region, which would render the kinase domain non-functional. We were unable to observe an endogenous truncated “X2” protein by western analysis, but this may be because of low abundance and/or lack of reactivity to our antibodies. Our work suggests that the X2 protein isoform is unstable and that the PTC in exon 9 renders RIOK3 X2 variant mRNA a target of the NMD machinery. However, even as a short-lived protein the putative X2 protein isoform could have a role in slowing IFNB signaling, e.g., by competitively interfering with interprotein interactions that normally constitute essential parts of the IFN signaling pathway(s). Either possibility suggests that RIOK3 expression could be regulated qualitatively and/or quantitatively at the level of alternative splicing in order to fine-tune the host response to infection (see Figure 6).

Regulation at the level of alternative splicing is not uncommon among innate immune effector proteins [49,50,51,52,53,54]. For example, alternative splice variants of the inhibitor of κB kinase ε (IKKε) are translated and function as dominant negative inhibitors of IRF3 or NF-κB activation [53]. Similarly, TBK1 is alternatively spliced during SeV infection to produce an alternatively spliced isoform that shuts down virus-induced IFNB production [54]. Additionally, many transcripts originally thought to encode truncated proteins have since been characterized as NMD targets [55]. It is estimated that most human genes are alternatively spliced and about one-third of alternative transcripts contain PTCs, rendering them potential NMD targets [5,7]. NMD is commonly recognized as a mechanism to degrade misspliced or aberrant transcripts in order to protect the cell from deleterious protein isoforms. NMD is also involved in regulation of gene expression by coupling alternative splicing with nonsense-mediated decay [55]. 

In conclusion, we show that the transcript encoding RIOK3, an incompletely understood immune regulatory protein, can be rendered nonfunctional by an alternative splicing event that is triggered by viral infection or other innate immune stimuli. Our data suggest that expression of the alternatively spliced isoform of RIOK3 may be directly antagonistic to the downstream immune responses, and may be a mechanism by which cells prevent excessive immune response. Future studies will aim to identify which cis- and trans- splicing factors are involved in the alternative 5’ splice site selection event that leads to RIOK3 X2 variant production, and whether the truncated X2 isoform is functional. Since roles for RIOK3 have also been postulated in cancer and the tumor microenvironment [56,57], the regulation of RIOK3 splicing represents a potentially compelling area of study in the realm of host–virus interactions as well as other areas of immune biology and medicine.

## Figures and Tables

**Table 1:** Spliced Expressed Sequence Tags (ESTs) that contain RIOK3 X1 and X2 variant splice junctions.

EST	GENOME	TISSUE
BQ311197 X1	human	breast
BF134913 X1	mouse	mammary metastatic tumor tissue
BI156659 X1	mouse	gross tumor tissue
BE308968 X1	mouse	gross tumor tissue
DC420826 X2	human	uterus
CA317387 X2	mouse	fetal brain tissue
CA980650 X2	mouse	pooled embryonic limb
DB184699 X1/X2	human	liver tumor

## Data Availability

Not applicable.
